# Development and validation of a machine learning model to identify individuals at high risk for psychotic disorders using medical record data

**DOI:** 10.1186/s12888-026-07846-z

**Published:** 2026-02-10

**Authors:** Ben J. Marafino, Andrea H. Kline-Simon, Icelini Stavers-Sosa, David J. Cronkite, Lawrence D. Gerstley, Cimone Durojaiye, Ann Kelley, Linda Kiel, Arvind Ramaprasan, David S. Carrell, Robert B. Penfold, Matthew E. Hirschtritt

**Affiliations:** 1 Division of Research, Kaiser Permanente Northern California, Pleasanton, CA USA; 2 Department of Health Systems Science, Kaiser Permanente Bernard J. Tyson School of Medicine, Pasadena, CA USA; 3 Department of Psychiatry, Kaiser Permanente Oakland Medical Center, Oakland, CA USA; 4 Department of Psychiatry and Behavioral Sciences, University of California, San Francisco, San Francisco, CA USA; 5 Kaiser Permanente Washington Health Research Institute, Seattle, WA USA

**Keywords:** Psychosis, Prediction model, Electronic health records, Feature extraction, Gradient boosting, Elastic net

## Abstract

**Background:**

Reducing the duration of untreated psychosis among individuals with early psychosis is associated with improved clinical outcomes and decreased long-term impairment. However, timely identification of individuals at high risk for psychotic disorders in routine clinical practice is challenging, and many individuals are only identified several years following psychotic-symptom onset. This study aimed to leverage comprehensive electronic medical records to develop and validate a machine learning model to identify individuals at high risk of conversion to a psychotic-spectrum disorder (PSD).

**Methods:**

This was a cross-sectional, retrospective analysis of electronic health record (EHR) data consisting of clinician free-text documentation and structured data (i.e., age, sex, race/ethnicity, psychiatric diagnoses, encounter modality, and department) among 406,268 Kaiser Permanente Northern California members aged 15–29 years with ≥ 1 primary-care encounter between 2017 and 2019 (~ 1,694,531 encounters). Patients with a new-onset PSD were distinguished from those without a diagnosis if they had ≥ 1 PSD diagnosis within 12 months following the index primary care encounter. The prediction models were developed using cross-validation with the gradient boosting and elastic net algorithms on features extracted from notes, and validated in a random test set.

**Results:**

A gradient-boosting model including text features model yielded the highest area under the curve (AUC 0.827 [95% CI: 0.799 to 0.853]), outperforming an elastic-net model (AUC 0.791 [95% CI 0.760 to 0.821]) and a gradient-boosting model that incorporated only discrete variables (AUC 0.610 [95% CI 0.595 to 0.626]). Model performance was similar across subgroups by sex, age, and race/ethnicity. However, all models exhibited suboptimal calibration, with predicted probabilities systematically underestimating observed PSD risk. Increasing the ratio of PSD cases to non-cases improved discrimination, but worsened calibration. Further, predicted probabilities of developing a PSD compressed with imbalance, causing abrupt metric drops at higher thresholds.

**Conclusions:**

This study suggests that individuals at elevated risk of developing a PSD may be identified from a general clinical population using a machine-learning model trained on routine clinical documentation and structured EHR data. However, the low incidence of PSDs led to suboptimal calibration. Future studies may restrict prediction to populations with higher PSD incidence, such as mental health clinics, to improve model calibration.

**Clinical trial number:**

Not applicable.

**Trial registration:**

Not applicable.

**Supplementary Information:**

The online version contains supplementary material available at 10.1186/s12888-026-07846-z.

## Background

Psychotic spectrum disorders (PSDs) represent a significant public health concern, with substantial personal and societal impacts, including functional impairment, reduced quality of life, elevated healthcare costs, and increased mortality rates compared to the general population [1–3]. Early identification and intervention are critical, as delays in treatment are associated with poorer outcomes, including prolonged symptom severity and greater neurobiological deterioration [4]. The duration of untreated psychosis (DUP) is a key modifiable prognostic factor, with shorter DUP generally linked to higher remission rates and neuroprotective effects [5, 6]. However, routinely and accurately identifying individuals at risk for developing a psychotic disorder remains challenging, particularly in primary care settings, which is often the first point of contact for individuals experiencing recent-onset psychotic symptoms [7].

Recent advances in natural language processing (NLP) and machine learning hold the potential to predict the onset of mental health disorders, thereby allowing for early and tailored interventions. Multiple studies have sought to leverage patient-generated speech and writing to predict psychotic disorders, some with impressive preliminary accuracy rates [8]. However, routinely collecting such data outside of a research study may be challenging, especially in large, population-based settings. In that context, routinely collected electronic health record (EHR) data is another, more readily available source of NLP data among health care-seeking individuals. In fact, numerous studies demonstrate the utility of NLP-based prediction models for various psychiatric conditions [9, 10].

Comparatively few of these EHR-based studies have applied these methods to identify individuals at risk for PSDs. For instance, using data from patients in the South London and Maudsley NHS Foundation Trust, researchers refined a previously validated, EHR-derived 5-item psychosis risk calculator to include 14 additional predefined NLP predictors (e.g., disturbed sleep, poor insight, paranoia) [11]. The resulting model was externally validated among patients receiving mental health services and demonstrated a 7.6% increase in predictive accuracy over the original 5-item model. In a more recent study using EHR data from Danish psychiatric clinics, researchers sought to predict 5-year transition risk to schizophrenia or bipolar disorder by developing and externally testing a model that integrated medication lists, diagnoses, and the text of clinical notes [12]. Using elastic net regularized logistic regression and extreme gradient boosting (XGBoost) models, the best-performing model yielded an area under the receiver operating characteristic curve (AUROC) for risk of progression to schizophrenia of 0.80 (95% CI 0.79 to 0.81), a specificity of 96.3%, a sensitivity of 19.4%, and a positive predictive value (PPV) of 10.8%.

However, to our knowledge, while at least one previous study in the United Kingdom was able to successfully predict incident psychosis in the primary care setting using routinely-collected data [13], no previously published studies have attempted to use machine learning to leverage unstructured clinical note text to predict progression to a psychotic-spectrum disorder in an undifferentiated general population. This study aimed to develop and validate an early warning system for PSD in the form of a machine learning model using NLP-derived features from unstructured clinical text and structured EHR data to predict the 12-month risk of incident PSD among young adults (aged 15 to 29 years) receiving care within Kaiser Permanente Northern California. By leveraging the comprehensive data from this integrated healthcare system, we sought to create a prediction model capable of real-time risk stratification, providing front-line clinicians with actionable insights to guide early assessment and intervention.

## Methods

This study was approved as minimal risk by the Kaiser Permanente Northern California (KPNC) Institutional Review Board. The detailed study protocol and cohort characteristics for this project have been published elsewhere and are summarized here [14]. Of note, the initial study protocol proposed incorporating data from both KPNC and a second Kaiser Permanente region (Washington) and text from secure patient messages; in contrast, the study described here exclusively applied data from the KPNC EHR, consisting of free-text clinician documentation and structured EHR-derived variables (described below). Where applicable, the reporting of this study conforms to the TRIPOD statement [15].

### Study design and cohort selection

KPNC is an integrated health-care delivery system that serves ~ 4.5 million members in Northern and Central California. Member characteristics (i.e., age, sex, race/ethnicity, socioeconomic indicators) are largely representative of the local and statewide population [16]. For this retrospective analysis, index encounters for the members in our study cohort comprised all their primary-care encounters (i.e., family practice, internal medicine, pediatrics, preventive medicine, and geriatrics) occurring between January 1, 2017 and December 31, 2019. We refer to these index encounters as *screen-eligible encounters (SEEs).*

The inclusion criteria were: (1) age 15–29 years; (2) ≥ 2 years of continuous plan enrollment; and (3) no history of an ICD-10-CM diagnosis for a PSD prior to the SEE. Members with no prior SEEs or for whom the SEE coincided with the first PSD diagnosis were excluded. There were no other exclusion restrictions, including exclusions for the primary complaint associated with the encounter. The prediction target (outcome) was an ICD-10-CM PSD diagnosis within 12 months following a SEE. To align with the intended operational use of the predictive model (i.e., to provide time-updated risk estimates) and to prevent possible bias by restricting the data to include only the SEE before the PSD diagnosis (which is not knowable at prediction time), we allowed members in our study cohort to have multiple SEEs, with a new prediction generated at each SEE.

### Feature engineering

Features for prediction models were derived from both structured EHR data and unstructured clinical text. There were six structured features which included patient demographics (i.e., age, sex, race, and ethnicity), clinical department, and encounter type (i.e., in-person, phone, or video). For unstructured data, natural language processing was used to extract and concatenate text features from clinical notes recorded in the 2 years preceding (but not including) the SEE, to avoid data leakage. Unigrams and bigrams were extracted from clinical text, along with keywords mapped from structured data concepts, as previously described [14]. Term presence and frequency were computed separately for the training and test sets, transformed using term frequency-inverse document frequency (TF-IDF) weighting, and combined with structured variables to generate the predictor set for model development.

### Machine learning model development and evaluation

We developed gradient boosting and elastic net models, which were cross-validated (using nested 10-fold CV) and trained on 80% of the cohort, then validated on the remaining 20%, which was treated as a held-out test set. 95% confidence intervals for performance metrics in the 20% test set were obtained via repeated random resampling. To prevent data leakage, all data splits were performed randomly at the patient level rather than at the encounter level, ensuring that no individual contributed data to both training and validation sets. Gradient boosting models were implemented using LightGBM with hyperparameters tuned via Optuna. Elastic net models were implemented using scikit-learn with grid search used to optimize the regularization parameters alpha (controlling overall regularization strength) and l1_ratio (controlling the balance between *L*_*1*_ and *L*_*2*_ penalties). Individual feature contributions were interpreted using SHapley Additive exPlanations (SHAP) analysis [17]. Model performance was assessed primarily via the area under the receiver operating curve (AUROC) and calibration plots, as well as sensitivity, specificity, positive predictive value, and related metrics. To assess the stability and performance of model discrimination and calibration, as well as to simulate development and validation in varying settings, we conducted sensitivity analyses by up-sampling PSD cases to non-cases at ratios from 1:1 to 1:128 and evaluating the performance of the resulting models in these simulated environments.

## Results

### Cohort characteristics

From January 1, 2017, through December 31, 2019, we identified 1,694,531 screen-eligible encounters among 406,268 KPNC members aged 15 to 29 years at cohort entry. Among these encounters, 3,162 (0.19%) were followed by a psychotic spectrum disorder (PSD) diagnosis within one year. The study population had a mean age of 20.1 years (standard deviation [SD]: 4.9 years) at cohort entry, with 46% being male. For individuals who developed PSD (cases), median time from cohort entry to PSD diagnosis was 780 days (interquartile range, 480 to 1042 days). The cases had a mean of 5.5 encounters (SD: 5.6 encounters) during follow-up, compared to 4.2 (SD: 4.1 encounters) among non-cases.

### Gradient boosting discrimination and calibration

The prediction models incorporated 3,141,556 features derived from the KP HealthConnect electronic health record system, comprising 3,141,550 unstructured text features extracted from clinical notes using natural language processing and 6 structured features. The gradient boosting model achieved superior discriminative performance with an area under the receiver operating characteristic curve (AUC) of 0.827 (95% CI 0.799 to 0.853), compared to the elastic net model’s AUC of 0.791 (95% CI 0.760 to 0.821). The gradient boosting model including text features significantly outperfomed a gradient boosting model trained with the structured features alone, which achieved an AUC of 0.610 (95% CI 0.595 to 0.626). Model performance across demographic subgroups was substantially similar, with no significant differences across sex (AUC 0.795 for females vs. 0.820 for males) nor across age (0.802 for age at cohort entry < 18 vs. 0.829 ≥ 18) nor across race or ethnicity. However, model calibration was suboptimal, with predicted probabilities systematically underestimating the true risk of PSD across most risk strata. Applying techniques including class weighting during training, Platt scaling, or incorporating an *α*-balanced focal loss term into the loss function, did not materially improve calibration.

**Fig. 1:**
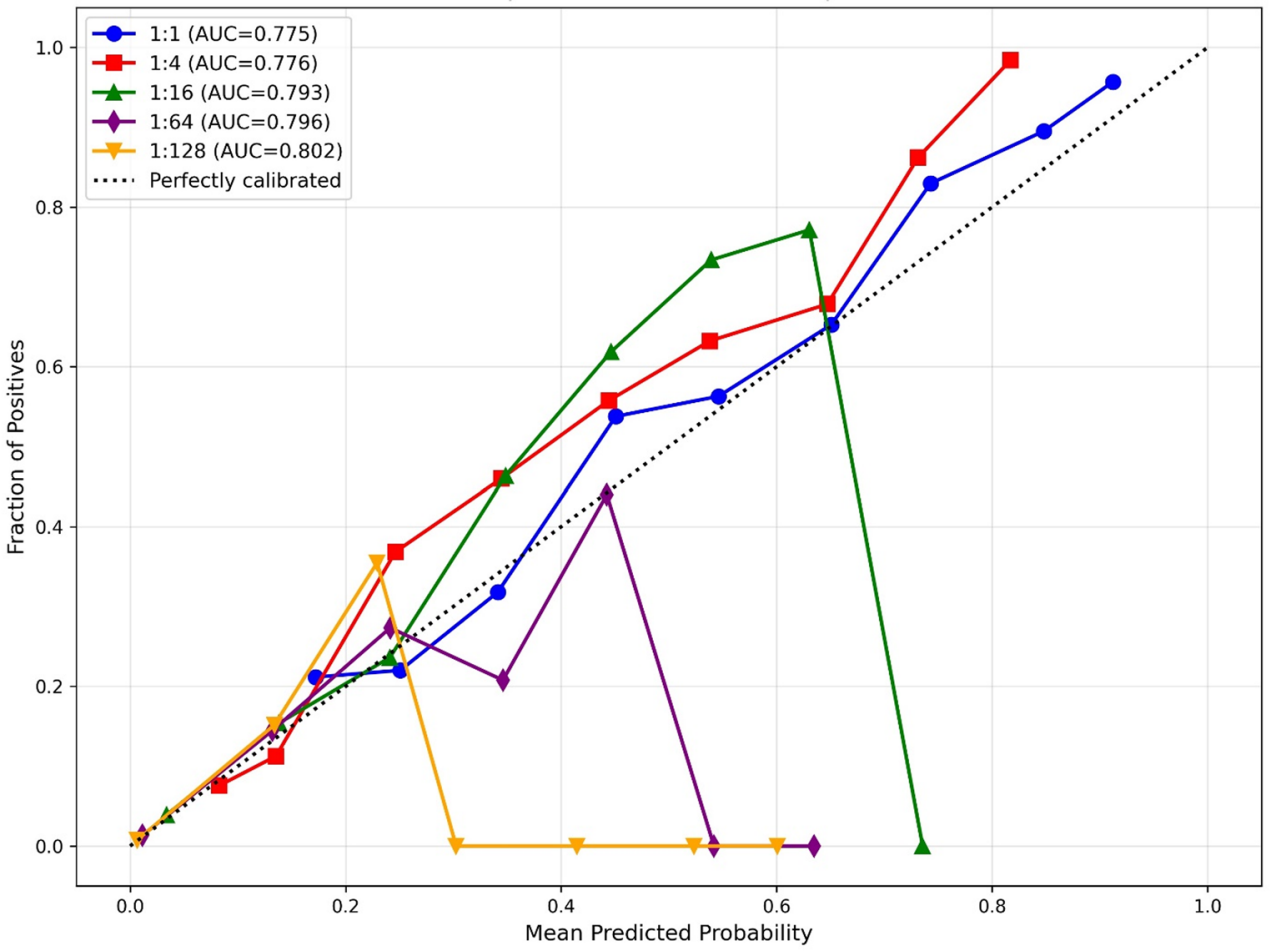
Calibration curves for five gradient boosting models trained with different case-to-non-case ratios, demonstrating a trade-off between model discrimination (AUC) and calibration as this ratio increases

Moreover, there was also a marked trade-off between the class distribution of cases and non-cases, and calibration performance (Fig. 1). While model discrimination improved as the case-to-non-case ratio (class ratio) approached the true class distribution (AUC increasing from 0.775 at 1:1 to 0.802 at 1:128), calibration substantially deteriorated. Models trained with balanced classes (1:1 and 1:4 class ratios) demonstrated more stable calibration curves that tracked closer to perfect calibration across most predicted probability ranges, though they still systematically underestimated risk. In contrast, models trained with class imbalance ratios approaching the true class distribution (1:64 and 1:128) appeared miscalibrated, with the fraction of positives dropping suddenly to zero for predicted probabilities above 0.4–0.6, despite achieving better discrimination. The 1:16 ratio appeared to represent a middle ground, maintaining reasonable discrimination (AUC 0.793) while exhibiting some calibration instability at higher predicted probabilities.

**Fig. 2:**
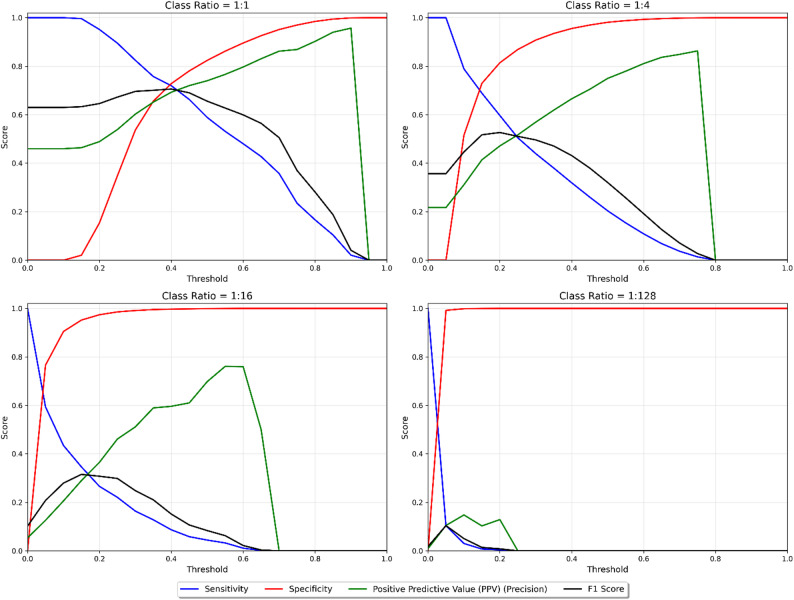
Sensitivity, specificity, precision, and F1 score as a function of decision threshold for four gradient boosting models trained with different case-to-non-case ratios, demonstrating several fundamental tradeoffs between these metrics as the threshold was varied

For the gradient boosting models, the relationship between sensitivity, specificity, and precision (positive predictive value) varied across decision thresholds and class imbalance ratios (Fig. 2). At balanced class ratios, the tradeoff between sensitivity and specificity was symmetric, and exhibited smooth transitions across the full range of thresholds. While the inverse relationship between sensitivity and specificity persisted across all ratios, higher sensitivity was associated with lower specificity as class imbalance increased. The sensitivity-precision tradeoff was most evident at higher imbalance ratios. At balanced class ratios, moderate sensitivity values corresponded with stable precision values. However, at imbalance ratios approaching the true class distribution, sensitivity and precision showed a stronger inverse relationship, with precision declining to zero beyond certain threshold values.

Figure 2 depicts the sensitivity, specificity, positive predictive value, and F1 score as a function of the decision threshold for the gradient boosting model in the setting of simulated class ratios of 1:1, 1:4, 1:16, and 1:128. Several findings are notable. With increasing class imbalance, the positive predictive value progressively deteriorated, with peak values declining from approximately 0.95 at a class ratio of 1:1 to 0.15 at 1:128. As the decision threshold was varied, the threshold maximizing F1 score shifted leftward with increasing imbalance, from approximately 0.4 at 1:1 to 0.1 at 1:128. As expected, a greater degree of imbalance compressed the range of predicted probabilities output by the model, as evidenced by the abrupt drop to zero in all metrics beyond thresholds of approximately 0.8 for 1:4 and 0.6 for 1:16 ratios.

**Fig. 3:**
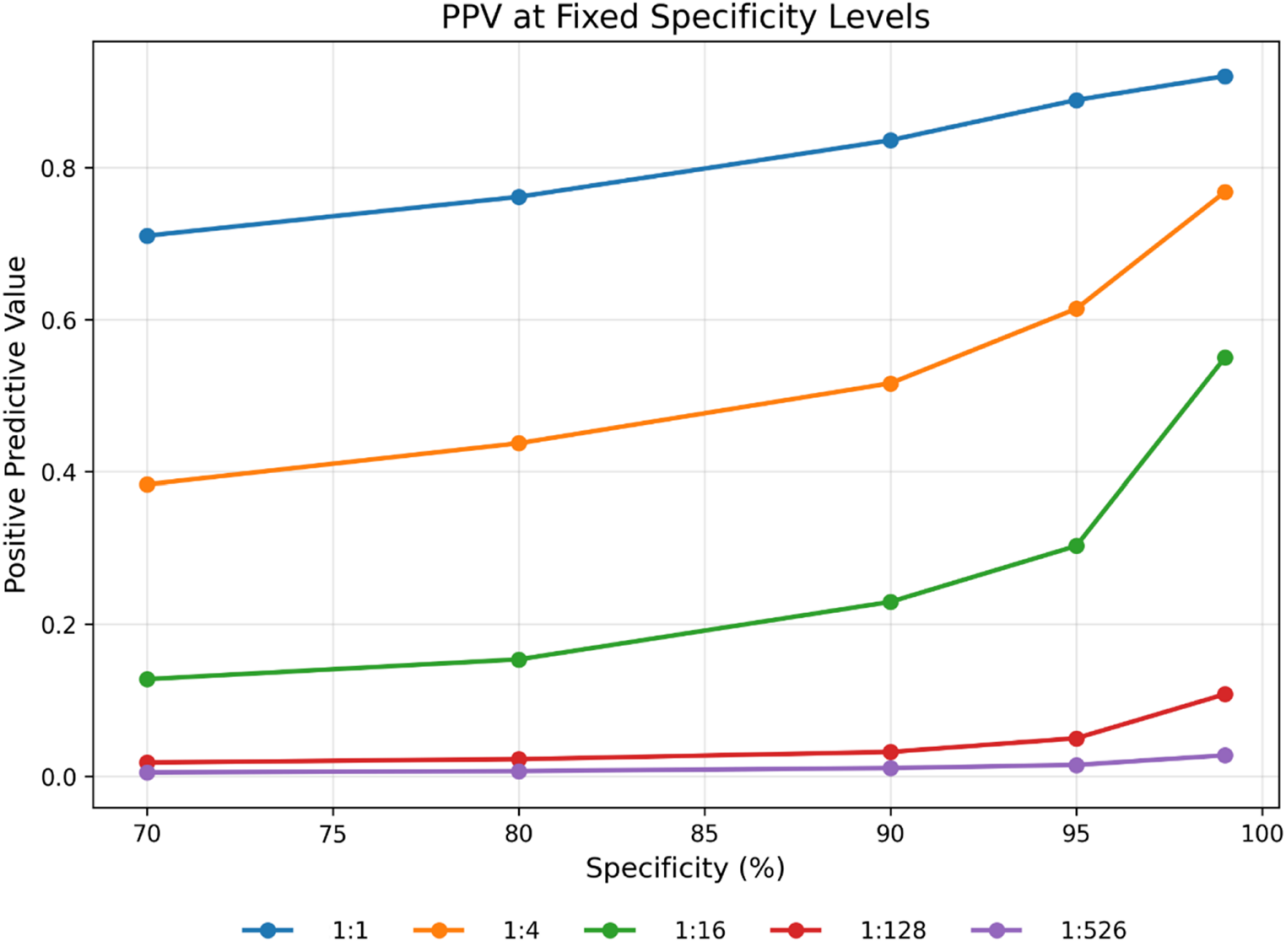
The positive predictive value (PPV) of the gradient boosting model (y-axis) across class ratios as a function of the specificity (x-axis) which ranged from 70% to 99%. The decision threshold was selected to fix the specificity at the given values

**Fig. 4:**
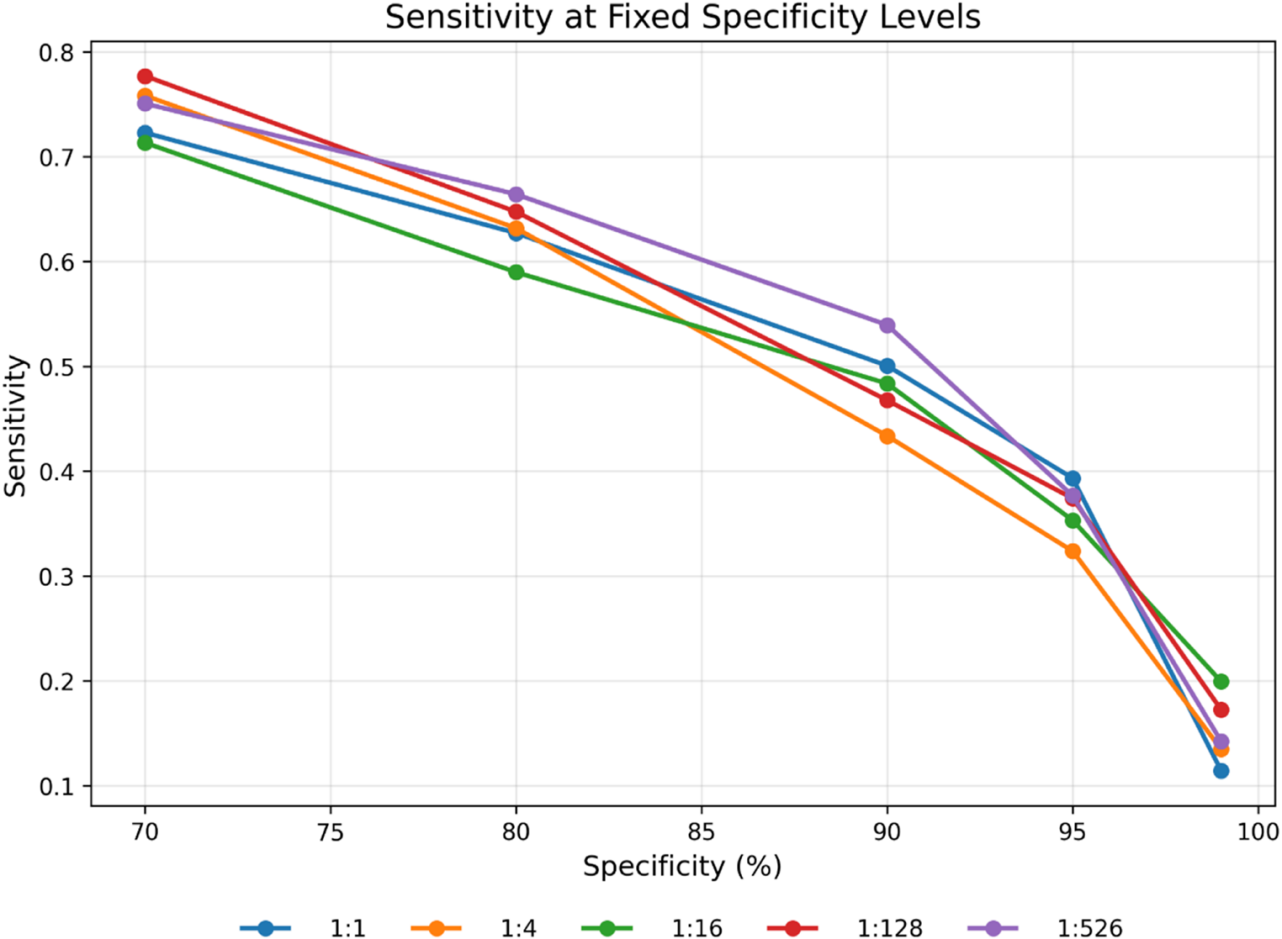
The sensitivity of the gradient boosting model (y-axis) across class ratios as a function of the specificity (x-axis) which ranged from 70% to 99%. The decision threshold was selected to fix the specificity at the given values

Figure 3 and Supplementary Table 1 show the gradient boosting model’s performance at fixed specificity levels across different case-to-non-case ratios. The positive predictive value (PPV) deteriorated with increasing class imbalance. At balanced sampling (1:1), PPV ranged from 0.71 at 70% specificity to 0.92 at 99% specificity. However, as the ratio approached the true population prevalence, PPV declined substantially: at 1:16, PPV ranged from 0.13 to 0.55; at 1:128, from 0.03 to 0.10; and at the native prevalence (1:526), PPV remained below 0.03 across all specificity levels. In contrast, sensitivity remained substantially similar across all class ratios, as would be expected from theory, and decreased monotonically with increasing specificity (Fig. 4).

### Model interpretability

**Fig. 5:**
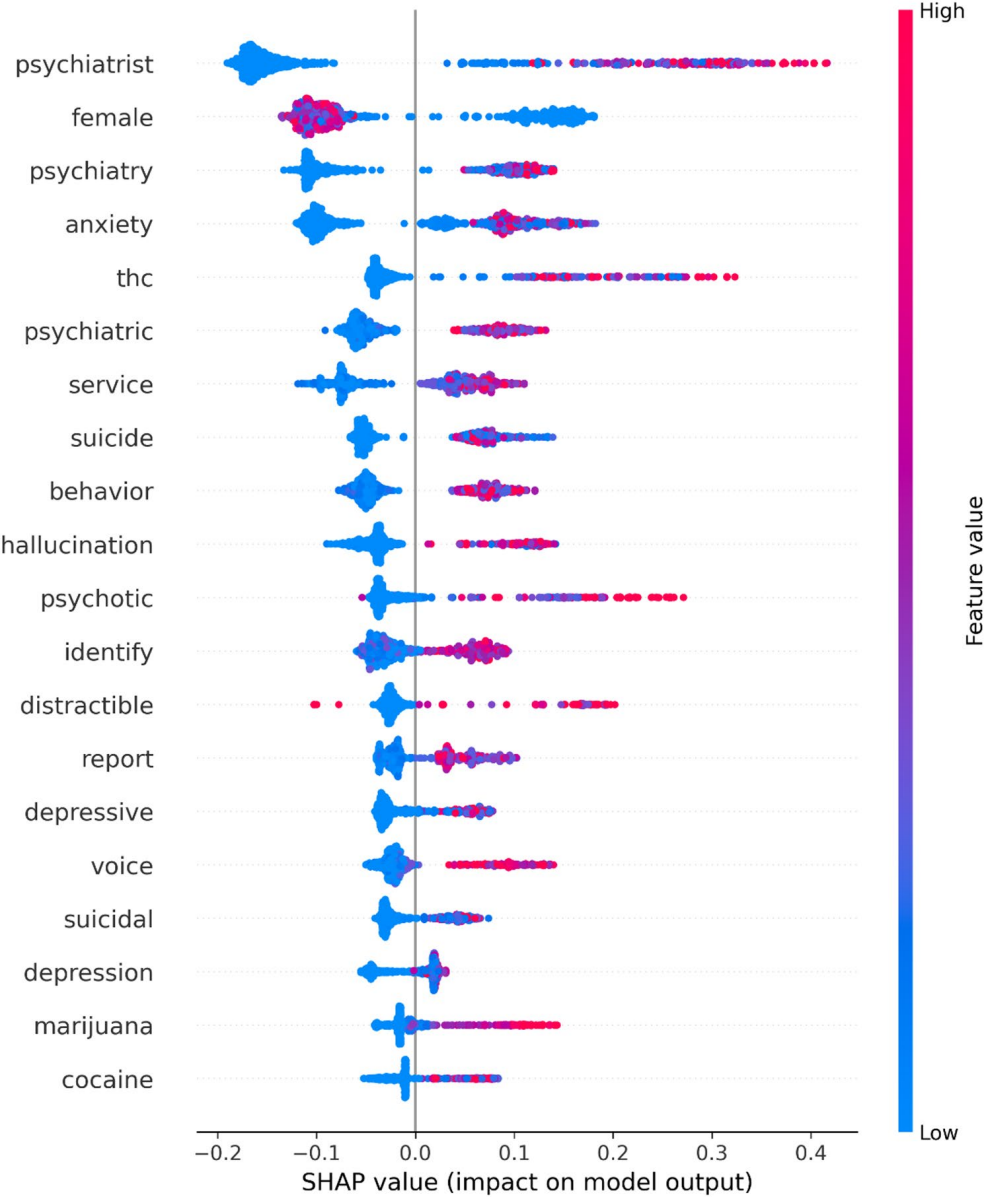
SHapley Additive exPlanations (SHAP) beeswarm plot displaying the top 20 most important text-derived features for the gradient boosting model using an outcome-stratified random sample of 1,000 encounters. Each point represents an individual encounter, with position on the x-axis indicating the feature’s impact on the predicted probability of PSD (SHAP value) and color representing the feature value (pink/red for high values, blue for low values). Features are ranked by mean absolute SHAP value

The relative importance and directional impact of the top 20 features on predictions are shown in a SHAP beeswarm plot (Fig. 5). The feature “psychiatrist” demonstrated the greatest influence on model output, with SHAP values spanning from approximately − 0.2 to 0.4 on the absolute risk scale, indicating substantial variability in its predictive impact across patients. Notably, several psychiatric symptom and service-related features (e.g., “anxiety”, “psychotic”, “psychiatric,” “psychiatrist”) exhibited wide distributions of SHAP values, suggesting more heterogeneous effects on the ensuing predictions. However, these features related to psychiatric service utilization did not appear to be major drivers of prediction; in a sensitivity analysis with these features ablated, the gradient-boosting model achieved an AUC of 0.801 (95% CI 0.772 to 0.830) which was not significantly different compared to an AUC of 0.827 (95% CI 0.799 to 0.853) for the model including all features. Similarly, the feature “thc” (e.g., “delta-9-thc”) was also associated with a wide distribution of SHAP values. In contrast, other substance-related features such as “cocaine” and “marijuana” exhibited relatively tight clustering of SHAP values near zero, indicating more consistent but modest contributions to model predictions. The color gradient implies that higher feature values (indicated in pink to red) for terms like “psychiatrist,” “female,” and “anxiety” were associated with higher SHAP values (generally pushing model predictions in the positive direction) while features such as “distractible” showed more complex patterns with high feature values distributed across both positive and negative SHAP values. When also including structured features, male sex and Black race emerged as the 3rd and 6th most important features, respectively, as assessed by their mean absolute SHAP values while the remaining top 20 features remained substantially unchanged. The accumulated local effect (ALE) plots for these top features are depicted in Supplementary Fig. 1, while their odds ratios for the elastic net model are given in Supplementary Table 2.

## Discussion

In this study, we successfully developed and validated machine learning models leveraging natural language processing of clinical notes combined with structured EHR data to predict 12-month risk of conversion to PSD among adolescents and young adults in primary care settings. Our gradient boosting model achieved strong discriminative performance (AUC of 0.827), with the addition of text-derived features providing substantial improvement over structured features alone (AUC of 0.610). Furthermore, the model demonstrated face validity, with top predictive features including clinically meaningful terms derived from free text such as “psychiatrist,” potential prodromal symptoms of PSD (e.g., “anxiety”, “hallucination”, “distractible”, “voice”), and substance-related terms (e.g., “thc”, “cocaine”), aligning with established risk factors for psychotic disorders.

To the best of our knowledge, our study is among the first to develop a population-scale risk prediction model for conversion to psychosis in a large and undifferentiated cohort, following similar work in the United Kingdom in a primary care population [13], and potentially the first to incorporate features derived from primary care clinical notes as predictors. While several previous studies have built models to predict conversion to psychosis, these efforts have primarily focused on individuals already determined to be at high risk or already receiving specialty mental health services, and often had access to richer sets of features, including biomarkers (e.g., salivary cortisol levels) and structured clinical assessments of positive and negative symptoms [18–22]. In addition, some larger studies building similar models, while not focusing on high-risk individuals, used a case-control design, which necessarily limits generalizability to a broader population [23–25]. Altogether, our study demonstrates the feasibility of building discriminative risk stratification models for conversion to psychosis at the population level using routinely-collected clinical free-text data, but our findings also demonstrate the challenges associated with automated population-scale screening enabled by predictive modeling.

Despite strong discrimination, model calibration proved suboptimal at the native class prevalence of approximately 0.19%, with predicted probabilities systematically underestimating true risk across most strata. Our finding that calibration deteriorated as we approached the true class distribution while discrimination improved illustrates a well-recognized trade-off in predictive modeling: in extremely low-prevalence settings, models must choose between accurately ranking individuals by risk (discrimination) or providing reliable absolute risk estimates (calibration). This trade-off has long been recognized by practitioners, and recently, has been encountered in prior work building prediction models for other rare psychiatric outcomes, most notably suicide [26–31]. Overall, our findings underscore the importance of assessing calibration, which has been referred to as the “Achilles heel” of predictive analytics, when working towards eventual real-world model use [32]. Indeed, prior studies building prediction models for psychiatric outcomes have inconsistently reported calibration. A recent systematic review of such models found that while 271 (88%) of the 308 models analyzed reported discrimination metrics, only 68 (22%) reported calibration, of which only 36 presented a calibration plot [33].

Viewed from a clinical perspective, the low positive predictive values observed at the native prevalence may limit the feasibility of deploying models to routinely screen for psychosis risk in undifferentiated primary care populations consisting of “all-comers”. At the native class prevalence, positive predictive values remained below 3% across all notional risk cutoffs, implying that most individuals flagged would not subsequently develop PSD following a SEE. In practice, such operating characteristics would likely lead to substantial alert burden among either primary care clinicians and frontline staff, raising concerns around resource allocation and potential stigmatization.

Our findings highlight another fundamental challenge in developing prediction models for psychotic spectrum disorders in adolescents and young adults: the limited and ambiguous signal inherent in this population. The 15-to-29 age range encompasses a period of significant developmental change, potentially making it difficult for models to disambiguate normative adolescent experiences and “growing pains” from genuine prodromal symptoms of psychosis [34]. Additionally, substance use, particularly cannabis use, as evidenced by the surfacing of “thc” in the SHAP analysis, is common in this age group but does not necessarily indicate increased risk of conversion to PSD [35]. These ambiguous signals are likely compounded by our models’ reliance on NLP-derived features from clinical notes, which, while valuable, may not capture the nuanced clinical assessments required to differentiate between benign developmental phenomena and true psychosis risk.

Our study has both strengths and limitations. Strengths include a large and demographically diverse cohort of over 400,000 adolescents and young adults with near-complete data capture for both predictors and outcomes within an integrated healthcare system. This routine primary care setting enhances the ecological validity of our findings compared to prior studies conducted exclusively in specialty mental health settings, where individuals have already been identified as high-risk. Furthermore, our gradient-boosting model achieved strong discriminative performance (AUC of 0.827) for this rare outcome in this undifferentiated primary care population, substantially outperforming models using structured data alone. Finally, our study design also constitutes a strength. By oversampling the outcome, we were able to quantify the discrimination-calibration tradeoff and demonstrate how model performance may improve in higher-prevalence settings, such as in specialty psychiatry clinics or in primary care restricted to patients with preexisting mental health diagnoses.

Limitations, in addition to the suboptimal calibration observed at the native class ratio (1:526), include the absence of several data sources that could enhance risk stratification. We lacked access to structured, self-reported mental health screening instruments; specialized psychosis risk assessments; cognitive testing results; detailed family psychiatric history; and pharmacy-dispensing data. Moreover, our study captured only a narrow temporal window of the prodromal trajectory. With only up to three years of observation possible within our study period and a 12-month prediction window, we were unable to identify individuals with protracted prodromal periods and those whose symptoms preceded their enrollment in this study. In some cases, the psychosis prodrome can span many years, which would extend beyond our observation window. Moreover, our focus on the 15-to-29 age range, while capturing the peak incidence period, limits generalizability both to younger adolescents and to older adults who may present with late-onset psychotic disorders, which often have distinct clinical features and trajectories.

In light of these findings, future work should shift from population-wide screening toward more targeted clinical contexts. Although our models were trained in a general primary care population, psychosis remains a rare outcome in this setting, which constrains positive predictive value and calibration, and therefore limits clinical utility. Restricting prediction to enriched populations, such as patients already engaged in specialty mental health care, or to those flagged as potentially at-risk in primary care based on screening tools, clinician concern, or diagnosis codes, appears necessary to achieve clinically meaningful performance. In such enriched settings, these models may function more appropriately as a second-stage risk stratification tool rather than as a stand-alone screening test.

Second, it is still unclear how to best translate probabilistic predictions into clinically actionable outputs. Tiered decision-support tools, such as a “stoplight” framework (e.g., green/yellow/red risk zones based on multiple probability thresholds), could help clinicians and support staff interpret and act on model output. However, such approaches depend on reliable calibration in order to appropriately sort patients into tiers of risk. Our findings underscore that discrimination alone is insufficient for real-world implementation; improving calibration and characterizing its degradation under class imbalance in a given setting must be central to any future work aiming to predict not only conversion to psychosis but the onset of other mental health disorders.

Third, the downstream consequences of algorithmic labeling of individuals as at-risk for psychosis warrant investigation. While early identification is a clinical priority, such algorithmic labeling may carry unintended psychological, social, and ethical harms, particularly in low-risk primary care settings where such predictions may be unexpected or stigmatizing [36, 37]. These harms may be attenuated, though potentially more heterogeneous, in behavioral health contexts where psychiatric labels are already part of the clinical discourse. For example, individuals with paranoid or borderline personality traits may respond very negatively to being labeled at risk for psychosis, while others already engaged in ongoing psychiatric care may view such labeling as more acceptable. However, these hypotheses remain untested.

## Conclusions

This study suggests that individuals at elevated risk of developing a PSD can be identified within a general primary care population using a machine learning model trained on routine clinical documentation and structured EHR data. The trade-offs we encountered between model discrimination and calibration — owing to the low rate of conversion to PSDs — are not unique to this setting but rather reflect fundamental challenges in predictive modeling for rare outcomes. Future studies should emphasize calibration alongside discrimination and consider how the choice of modeling population (general vs. enriched) impacts both predictive performance and clinical utility. In this regard, our findings serve as both a demonstration of feasibility and a cautionary tale, highlighting the complexity of applying machine learning to predict emerging psychosis, as well as other rare mental health disorders, in routine care.

## Supplementary Information

Below is the link to the electronic supplementary material.

Supplementary Material 1

## Data Availability

The datasets analyzed during the current study are subject to institutional and ethical restrictions related to patient confidentiality and are therefore not publicly available. Access may be granted upon reasonable request to the corresponding author and contingent on applicable data use agreements and approvals.

## Abbreviations

AUC: Area under the curve
AUROC: Area under the receiver-operating curve
CI: Confidence interval
DUP: Duration of untreated psychosis
EHR: Electronic health record
ICD-10-CM: International Statistical Classification of Diseases and Related Health Problems 10th Revision—Clinical Modification
TF-IDF: Term frequency-inverse document frequency
NLP: Natural language processing
PSD: Psychotic-spectrum disorder
SEE: Screen-eligible encounter
SHAP: SHapley Additive exPlanations
