# Supplementary material for: Development and validation of a machine learning model to identify individuals at high risk for psychotic disorders using medical record data
Source: BMC Psychiatry. 2026 Feb 10;26:227. doi: 10.1186/s12888-026-07846-z (PMC12964962; doi:10.1186/s12888-026-07846-z)
Supplement: Supplementary file 1 — Supplementary Material 1 [file 12888_2026_7846_MOESM1_ESM.docx]

**Development and validation of a machine learning model to identify individuals at high risk for psychotic disorders using medical record data**

**Supplementary Information**

Corresponding author:

Ben J. Marafino Ph.D.

Kaiser Permanente Division of Research

4480 Hacienda Drive

Pleasanton, CA 94588

[ben.j.marafino@kp.org](mailto:ben.j.marafino@kp.org)

**Supplementary Table 1.** Number needed to evaluate (NNE) as a function of PSD case-non-case ratio and the operational risk cutoff (ORC) for the gradient boosting model at a target specificity, computed in the test set. The ORC determines threshold needed to yield the target specificity. The first three columns define a specific scenario, and the following three columns give the observed characteristics of the model-derived risk predictions under that scenario.

| PSD case-non-case  ratio | Target Specificity (%) | Top Risk Percentile for ORC | Resulting Operational Risk Cutoff (%) | Cases in Top Risk Percentile  (%) | Number Needed to Evaluate |
| --- | --- | --- | --- | --- | --- |
| 1:1 | 99 | 1 | 81.5 | 11.4 | 1.09 |
| 1:1 | 95 | 5 | 68.4 | 39.3 | 1.13 |
| 1:1 | 90 | 10 | 60.0 | 50.1 | 1.20 |
| 1:1 | 80 | 20 | 48.6 | 62.7 | 1.31 |
| 1:1 | 70 | 30 | 39.3 | 72.3 | 1.41 |
| 1:4 | 99 | 1 | 57.2 | 13.5 | 1.30 |
| 1:4 | 95 | 5 | 37.8 | 32.4 | 1.63 |
| 1:4 | 90 | 10 | 28.8 | 43.4 | 1.94 |
| 1:4 | 80 | 20 | 19.5 | 63.2 | 2.29 |
| 1:4 | 70 | 30 | 14.5 | 75.8 | 2.61 |
| 1:16 | 99 | 1 | 27.9 | 19.9 | 1.82 |
| 1:16 | 95 | 5 | 13.7 | 35.3 | 3.30 |
| 1:16 | 90 | 10 | 9.03 | 48.4 | 4.36 |
| 1:16 | 80 | 20 | 5.56 | 59.0 | 6.52 |
| 1:16 | 70 | 30 | 3.89 | 71.3 | 7.84 |
| 1:128 | 99 | 1 | 4.64 | 17.3 | 9.29 |
| 1:128 | 95 | 5 | 2.06 | 37.4 | 20.1 |
| 1:128 | 90 | 10 | 1.27 | 46.8 | 31.6 |
| 1:128 | 80 | 20 | 0.77 | 64.7 | 45.1 |
| 1:128 | 70 | 30 | 0.59 | 77.7 | 56.1 |
| 1:526 | 99 | 1 | 1.35 | 14.2 | 36.7 |
| 1:526 | 95 | 5 | 0.49 | 37.7 | 68.5 |
| 1:526 | 90 | 10 | 0.31 | 53.9 | 95.2 |
| 1:526 | 80 | 20 | 0.18 | 66.4 | 154.1 |
| 1:526 | 70 | 30 | 0.13 | 75.1 | 204.1 |

**Supplementary Figure 1.** Accumulated local effect (ALE) plot of the top 20 text features from gradient boosting models. The x-axis denotes the tf-idf value of that feature, while the y-axis denotes the ALE as measured on the probability scale for that feature, as a deviation from the mean prediction over the feature’s tf-idf range.

**
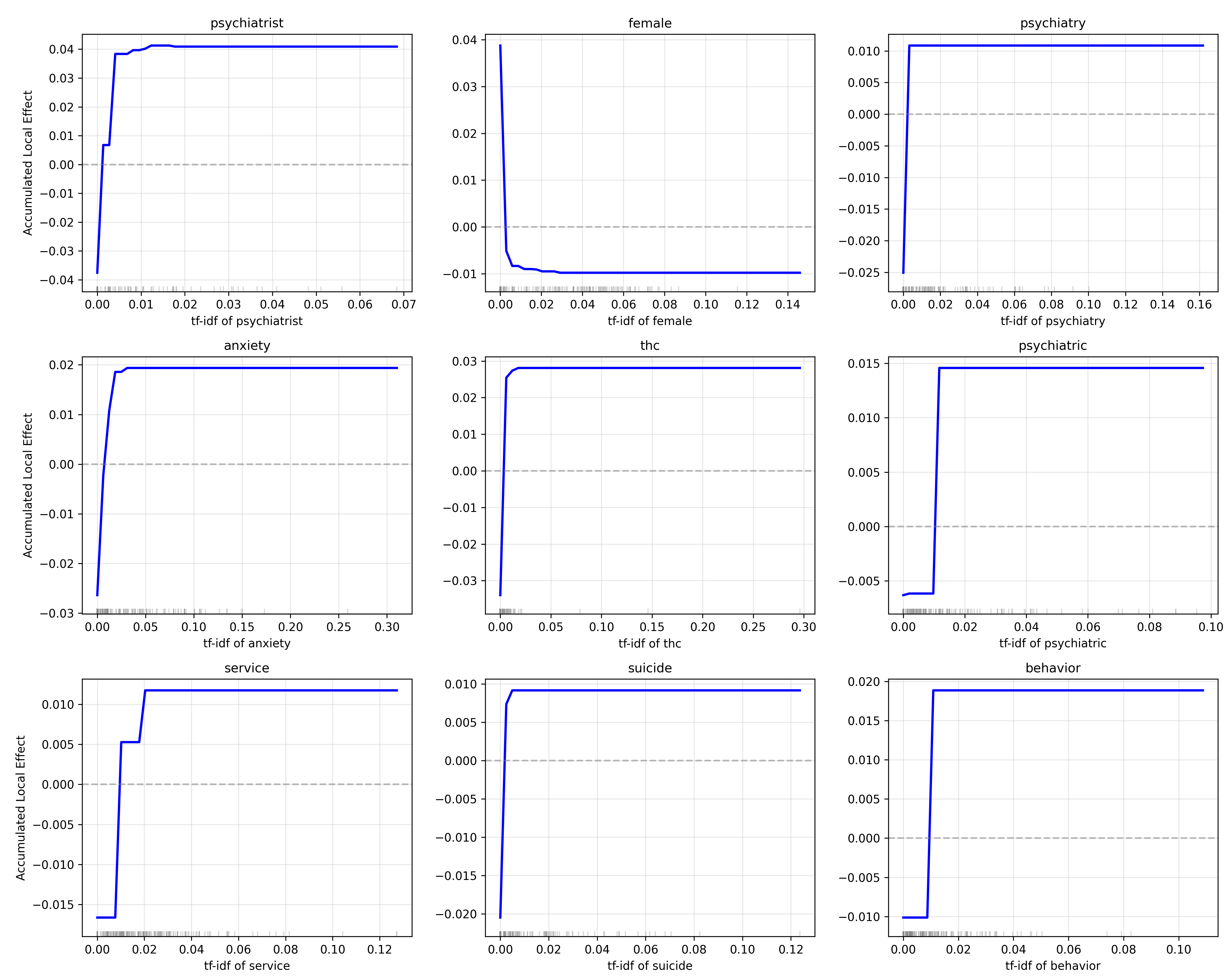
**

**Supplementary Figure 1.** Continued from above**.**

**
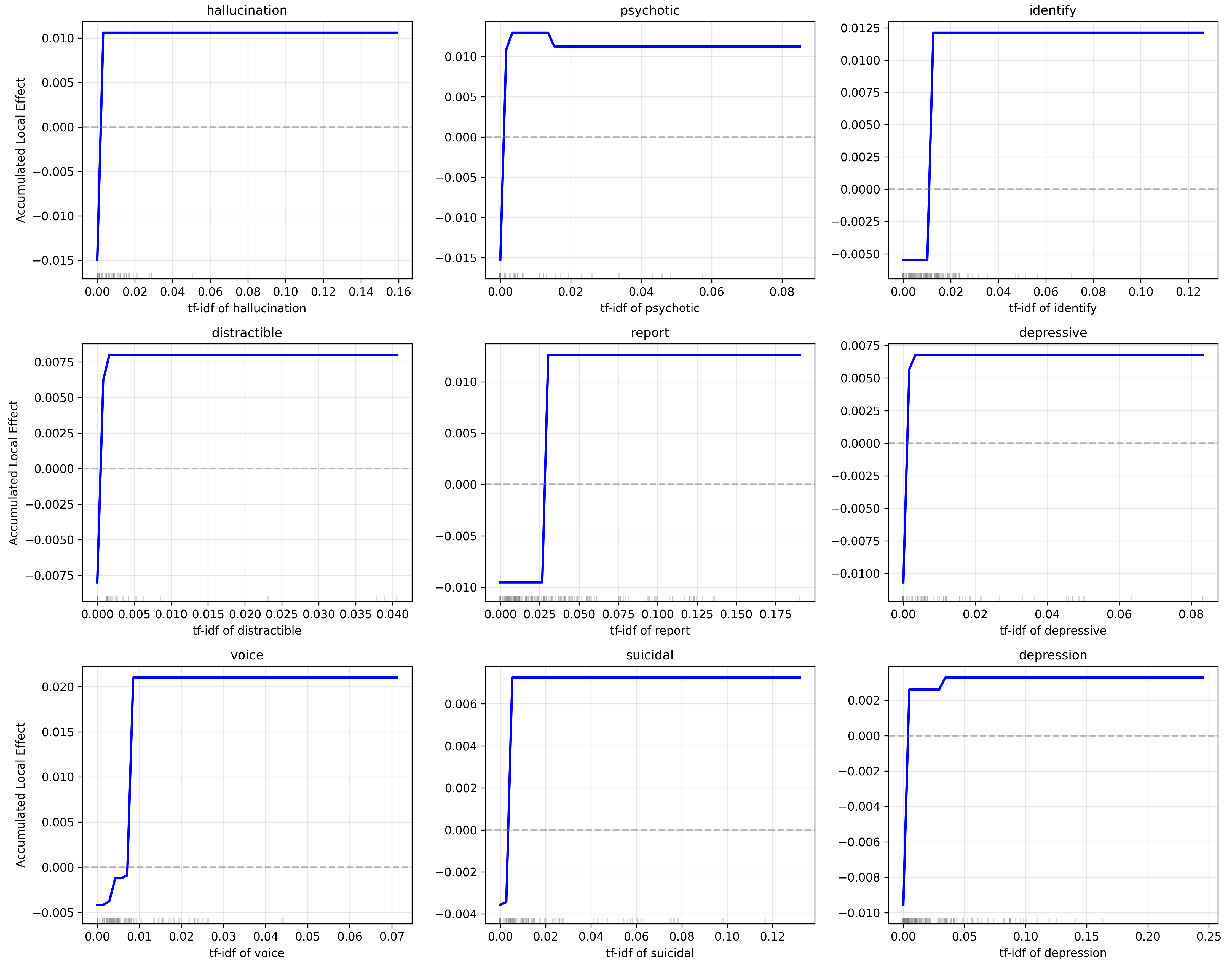
**

**
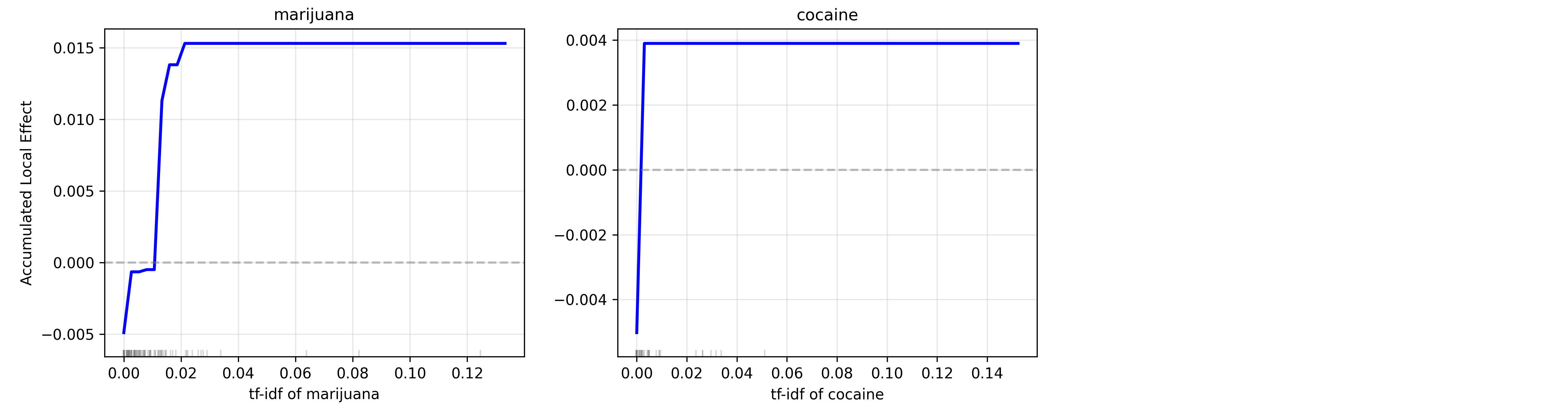
**

**Supplementary Table 2.** Top text features from the elastic net model, as ranked by the absolute value of their coefficient estimate, along with their standard errors and 95% confidence intervals. Features with a positive coefficient suggest increased risk, while features with a negative coefficient suggest decreased risk.

| Feature | Coefficient Estimate | Estimated Standard Error | Lower Bound of 95% CI for Estimate | Upper Bound of 95% CI for Estimate |
| --- | --- | --- | --- | --- |
| psychotic | 8.90 | 1.71 | 5.54 | 12.26 |
| preventive | -6.13 | 0.83 | -7.76 | -4.49 |
| molly | 6.11 | 0.59 | 4.96 | 7.26 |
| hallucination | 5.62 | 1.06 | 3.55 | 7.70 |
| sharp | -5.61 | 1.87 | -9.28 | -1.94 |
| hear | 5.60 | 1.11 | 3.44 | 7.77 |
| burn | -5.41 | 1.18 | -7.73 | -3.10 |
| fever | -5.24 | 0.39 | -6.00 | -4.48 |
| thc | 5.18 | 0.68 | 3.84 | 6.52 |
| depression | 5.07 | 0.29 | 4.51 | 5.64 |
| travel | -5.05 | 0.79 | -6.59 | -3.51 |
| basketball | -5.02 | 1.26 | -7.48 | -2.56 |
| holter | -4.88 | 1.85 | -8.51 | -1.25 |
| sad | -4.82 | 0.94 | -6.67 | -2.98 |
| paranoid | 4.81 | 2.39 | 0.12 | 9.50 |
| improve | -4.80 | 0.50 | -5.79 | -3.82 |
| suicidal | 4.48 | 0.56 | 3.39 | 5.57 |
| voice | 4.41 | 1.13 | 2.21 | 6.62 |
| denie | -4.31 | 2.00 | -8.23 | -0.38 |
| abuse | 4.18 | 0.59 | 3.02 | 5.34 |
| marijuana | 4.17 | 1.13 | 1.96 | 6.37 |
| psychiatrist | 4.09 | 1.04 | 2.06 | 6.13 |
| seasonal | -3.88 | 1.11 | -6.05 | -1.70 |
| anxious | 3.45 | 0.87 | 1.74 | 5.15 |
| identify | 2.13 | 0.69 | 0.77 | 3.49 |
